# Evaluating the Impact of an 8-Week Family-Focused E-Health Lifestyle Program for Adolescents: A Retrospective, Real-World Evaluation

**DOI:** 10.3390/nu17223509

**Published:** 2025-11-10

**Authors:** Susan Hulland, Oluwadurotimi Obatoki, Isabella Giardino, Caley Kirkman, Monica van Dam, Cecilia Airth, Lucy Quin, Brendan Goodger, Zoe E. Davidson

**Affiliations:** 1 Department of Nutrition, Dietetics and Food, Monash University, Notting Hill, Melbourne, VIC 3168, Australia; shul0006@student.monash.edu (S.H.); ooba0002@student.monash.edu (O.O.); igia0007@student.monash.edu (I.G.); ckir0012@student.monash.edu (C.K.); mvan0059@student.monash.edu (M.v.D.); 2 Better Health Company, Abbotsford, Melbourne, VIC 3067, Australia; cecilia.a@betterhealthcompany.org (C.A.); lucy.q@betterhealthcompany.org (L.Q.); 3 Central and Eastern Sydney Primary Health Network, Mascot, Sydney, NSW 2020, Australia; b.goodger@cesphn.com.au

**Keywords:** e-Health, overweight, obesity, adolescence, public health, behavior change

## Abstract

**Background/objectives:** Overweight and obesity in adolescents is a significant public health concern, yet limited interventions exist in Australia to promote healthy behavior change in families and young people. This retrospective, real-world evaluation aimed to describe the impact of an 8-week e-Health program (Think, Eat And Move, [TEAM]) on anthropometric, behavioral and wellbeing outcomes in adolescents. **Methods:** Eligible TEAM program participants were aged 13–17 years, resided in Central or Eastern Sydney, Australia, had overweight/obesity, were at risk of or had a chronic health condition and/or required healthy lifestyle support. Retrospective program data were used to assess the impact of TEAM on weight, height and BMI z-score, dietary intake, physical activity and wellbeing indices. **Results:** Of 567 registered participants, 313 completed the TEAM program and were included in the analysis (median age 14.4 years, 56.2% female). The median (interquartile range) BMI z-score reduced from 1.8 (1.4–2.2) pre-program to 1.6 (1.1–2.1) post-program (*p* < 0.001, *n* = 262). Significant improvements in health behaviors following the program were observed, including increased consumption of fruit, vegetables and water; reduced consumption of confectionery and take-away foods; increased days of physical activity; and reduced sedentary time. Significant positive changes were also observed in knowledge and wellbeing measures. **Conclusions:** Adolescents completing the TEAM program demonstrated clinically important changes in anthropometry and improved nutrition and physical activity behaviors. E-Health modalities for promoting behavior change should be considered in public health strategies for adolescents.

## 1. Introduction

Obesity in Australian adolescents is a significant public health concern, with profound implications for both immediate and long-term health outcomes [1]. In the 2022 National Health Survey, 29.6% and 31.7% of adolescents aged 12–15 years and 16–17 years, respectively, had overweight or obesity [1]. The causes of obesity are multifactorial, resulting from the complex interplay of environmental, genetic, psychological, dietary, activity and metabolic considerations contributing to energy imbalance and excess adiposity [2]. In adolescents, family, community and society also influence energy balance [3]. Specifically, key contributors to obesity include poor dietary habits including increased intake of sugar-sweetened beverages, physical inactivity and excessive recreational screen time [3]. Adolescents with overweight or obesity are at increased risk for a range of comorbidities, including hormonal imbalances such as insulin resistance and disruptions in reproductive and thyroid function, as well as cardiovascular alterations like hypertension, dyslipidemia and early signs of atherosclerosis [4,5,6,7]. These conditions not only affect immediate health but also contribute to the persistence of obesity into adulthood, amplifying the risk of chronic diseases such as type 2 diabetes, cardiovascular disease and certain cancers [7,8]. Approximately 80% of adolescents who live with overweight or obesity will maintain this status into adulthood [9,10], highlighting the urgency of targeted interventions to address this growing issue.

Despite the rising prevalence of obesity in adolescents, there are few public health lifestyle programs targeting this life stage. Adolescence (aged 13–19) is recognized as a critical stage of life, marked by dynamic brain development and interactions with social environments that shape a young person’s health and wellbeing [9,11,12]. It is a period of immense physiological and psychological transition towards independence. This transition is characterized by a range of significant changes. In addition to hormonal fluctuations, metabolic, cognitive, neural and behavioral developments contribute to the formation of an individual’s emotional and social identity [9].

During this period, young people also acquire the physical, cognitive, emotional, social and economic resources that form the foundation for lifelong health and wellbeing [9]. Developmental tasks, such as gaining independence, completing education, entering the workforce, forming relationships and engaging in their communities, are also underpinned by health and wellbeing [9]. The emergence of overweight and obesity during adolescence is associated with both immediate and long-term physical health risks, reduced quality of life and mental health challenges [7,13]. The interaction between these domains is complex and bidirectional, with mental health influencing health behaviors and vice versa [14]. Given the complex and unique nature of the adolescent period, finding effective and tailored solutions to support adolescents’ eating habits and lifestyle choices is crucial to reducing the prevalence of overweight or obesity.

In an attempt to reduce chronic disease and address modifiable risk factors amongst young people, the Australian National Preventative Health Strategy has endorsed the adoption of e-Health by governments and health care systems [15]. This has allowed for various digital programs that address obesity and mental health to be implemented across Australia [15]. E-Health is defined as “health services and information delivered or enhanced through the internet and related technologies” [16]. In contrast to traditional face-to-face sessions, e-Health demonstrates notable benefits such as increased engagement and reach and equitable access to healthcare [16,17]. Considering that approximately 91% of adolescents across Australia have easy access to smartphone applications and websites, programs delivered in this manner enhance the dissemination of educational content to adolescents [16,17].

There is emerging literature supporting the use of e-Health interventions for adolescent populations with scope for future research to advance digital modalities for health promotion [18]. A recent systematic review including 16 randomized clinical trials of interventions using smartphone apps and web platforms concluded that digital interventions have potential for shifting eating habits in adolescents; however, consideration is needed to enhance adherence and long-term engagement [19]. Similarly, an umbrella review of lifestyle interventions for children and adolescents delivered by e-Health and m-Health identified positive impacts of weight, diet and physical activity [20]. Notably, only 2 of the 25 included systematic reviews specifically targeted adolescents as opposed to children and adolescents. Considering the evidence specifically related to adolescents, there is minimal evidence regarding e-Health programs available for this age group in Australia, especially digital interventions that sit outside of the school environment [21].

The Think, Eat and Move (TEAM) program is a free 8-week e-Health program delivered to adolescents in the Central and Eastern Sydney Primary Health Network catchment area by the administering organization (Better Health Company, Abbotsford, Victoria, Australia). The program contains online coaching sessions and web-based education in nutrition and physical activity, focusing on empowering adolescents and their families/carers to make well-informed choices towards adopting healthier lifestyle habits [22].

The TEAM program offers a unique opportunity to assess the effectiveness of a community-based digital intervention for adolescents within an Australian context. By examining changes in anthropometric and health behavior outcomes, this study contributes to the growing evidence base for scalable, youth-focused health promotion strategies to improve health outcomes for current and emerging generations. This retrospective evaluation aims to address the following questions: what is the impact of the TEAM e-Health program on nutrition, physical activity and wellbeing outcomes among adolescents; and how do demographic factors relate to program engagement and withdrawal?

## 2. Materials and Methods

### 2.1. Study Design

This retrospective evaluation study collated anonymized data from adolescents aged 13–17 who participated in the TEAM program via Central and Eastern Sydney Primary Health Network (Australia) from 2018 to 2024. This study was approved by the Monash University Ethics Committee (Project Number: 45606). All participants’ caregivers provided written consent for their data to be used for research and evaluation in accordance with the administering organization’s privacy policy [23].

### 2.2. The TEAM Program

**Figure 1:**
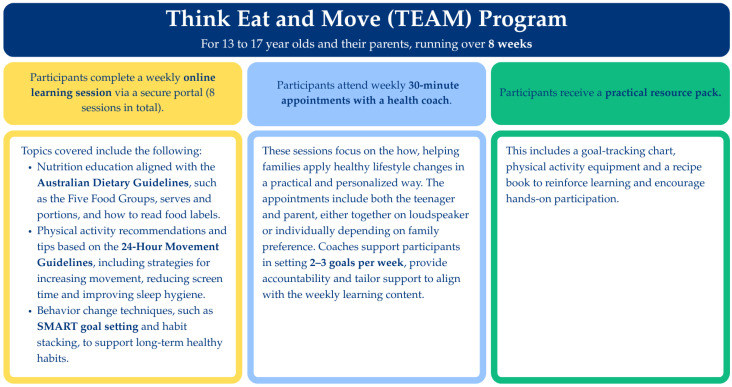
Overview of the key elements of the Think Eat and Move (TEAM) Program.

TEAM is an 8-week, online, family-focused program aimed at increasing nutrition knowledge, physical activity, confidence and wellbeing. Figure 1 provides an overview of the key elements of the program. TEAM was created by psychologists, dietitians and exercise physiologists using current evidence and Australian Government nutrition and physical activity guidelines. Following registration in the program for eligible participants, weekly appointments were scheduled with tertiary qualified health professional coaches. Additionally, participants received practical resources designed to help support and sustain healthy behavior change at home. The program was designed to be delivered flexibly to suit family life and provided online web-based modules and eight weekly 30 min coaching calls with the participants. The online modules consisted of interactive activities, narrated content, videos, animations and games with optional additional resources. The content of the modules focused on healthy lifestyle topics, including healthy eating, physical activity, screen time, sleep, mindfulness and goal setting. Behavior change strategies such as motivational interviewing and cognitive behavior therapy are used throughout the coaching sessions to facilitate the creation of personalized goals.

Between 2018 to 2024, there were three iterations of the TEAM program (herein Program 1, 2 and 3, respectively). These iterations reflected participant feedback and shifting perspectives on obesity. The iterations impacted participant eligibility criteria and program outcome measures (described below). In brief, a BMI  ≥  85th percentile was included in the eligibility criteria for Program 1 only, anthropometry outcomes were measured in Program 1 and 2 only, and self-esteem and body esteem were measured in Program 1 only. The delivery of TEAM remained constant across the programs.

### 2.3. TEAM Program Participants

To be eligible to complete the TEAM program, participants need to be aged 13–17 years and living in Central or Eastern Sydney. In addition, participants in Program 1 needed to be above the healthy weight range (BMI z-score  ≥  85th percentile), as defined by the Centers for Disease Control and Prevention (16), with one of the following: diagnosed with a chronic health condition; at risk of a chronic health disease (e.g., diabetes, heart disease and/or anxiety); or requiring healthy lifestyle support (healthy eating and/or physical activity). “At risk of chronic disease” was defined through referral by a health professional and/or self-reporting by the parent or carer based on advice or diagnosis received from their health professional. Participants were considered as “requiring healthy lifestyle support” in two ways. For those above a healthy weight, this criterion was automatically met. For participants not above a healthy weight, this was determined through a self-reported response during the registration process. Specifically, parents or carers were asked directly whether they felt their child required support to improve or maintain healthy lifestyle behaviors, based on their own observations or advice received from a health professional.

In Program 2 and 3, the BMI requirement was removed from the eligibility criteria. The decision to remove the BMI requirement from the eligibility criteria in Programs 2 and 3 was informed by both stakeholder feedback and internal program review findings. The change was made to reduce barriers to sign-up, referral and participation and to minimize the weight stigma associated with the program.

Young people living in Central or Eastern Sydney either self-referred to the program or were referred by a treating health professional using an online referral process. Written consent to participate in the program was provided by participants’ caregivers according to the administrative organization’s privacy policy [23]. Referring health professionals were given updates regarding the participants’ progress within the program.

### 2.4. Demographic Characteristics of Participants

Available demographic information included age, sex at birth, gender, Indigenous status, referring health professional occupation and postcode. Due to small numbers of participants identifying as Indigenous (<*n* = 5), this variable is not reported to avoid potentially identifying individuals. Socio-Economic Indexes for Areas (SEIFA) deciles were determined using postcode data. SEIFA was developed by the Australian Bureau of Statistics to rank geographic areas across Australia based on relative levels of socio-economic advantage and disadvantage [24]. These indexes are derived from data collected in the national Census, which occurs every five years. SEIFA scores reflect factors such as income, education, employment and housing characteristics. Importantly, SEIFA represents a summary of an area, not individual circumstances.

### 2.5. Outcome Measures

Outcome measures were self-reported by families via online surveys through the administering organization’s web platform. Table 1 provides details of information collected from the online surveys. Data was collected prior to commencing the program and immediately and 6 months after the program. Data from 6 months after the program were excluded from analysis, as there were minimal responses. In the case of withdrawals from the program, the date and reason (if provided) for withdrawal were recorded.

### 2.6. Analysis

Firstly, to describe the cohort, participants were grouped based on program completed (i.e., Program 1, 2 or 3) and on when they withdrew from the program. Withdrawal timepoints were categorized as (1) withdrew before pre-program: participant registered for the program but did not complete any pre-program surveys; (2) withdrew before the program: participant completed at least one pre-program survey but did not complete or attend any online or coaching sessions; (3) withdrew during the program: participant completed at least one pre-program survey and at least one online or coaching session but did not complete any post-program surveys; and (4) completed program: participant completed at least one pre-program and post-program survey and attended at least one online session or coaching session.

Assessment of the impact of the program on outcome measures included only participants who were categorized as completed program. Within the completers, individual survey results were included for analysis if both a pre- and post-program survey were available. Individuals who identified their social gender (*n* = 6) to be different than their biological sex were not included in the analysis of anthropometric data because it was not known if these individuals were undergoing gender-affirming care, which could influence results.

IBM SPSS Statistics version 30.0 (2024, Amronk, NY, USA) was used for statistical analysis of all quantitative data, with *p*-values < 0.05 being considered as statistically significant. Prior to conducting statistical analysis, continuous data was assessed for normality by Kolmogorov–Smrinov (*n* > 300) or Shapiro–Wilk (*n* < 300), histogram, normal and detrended Q-Q plots and box plots. Data was presented as mean ± SD if parametric or median (IQR, 25th–75th percentile) if non-parametric. Associations between withdrawal timepoint and age at start of program were assessed using the Kruskal–Wallis rank test. Other demographic characteristics were analyzed using the Chi-square test of independence. Anthropometric measurements for Programs 1 and 2 were mostly non-parametric and were analyzed using the Wilcoxon signed rank test. Outcome measures from surveys were analyzed using a Chi-square test of independence, excluding tests where the data had an expected cell count of <5 (20%). No measures were taken to adjust for confounders.

## 3. Results

### 3.1. Participant Characteristics

**Figure 2:**
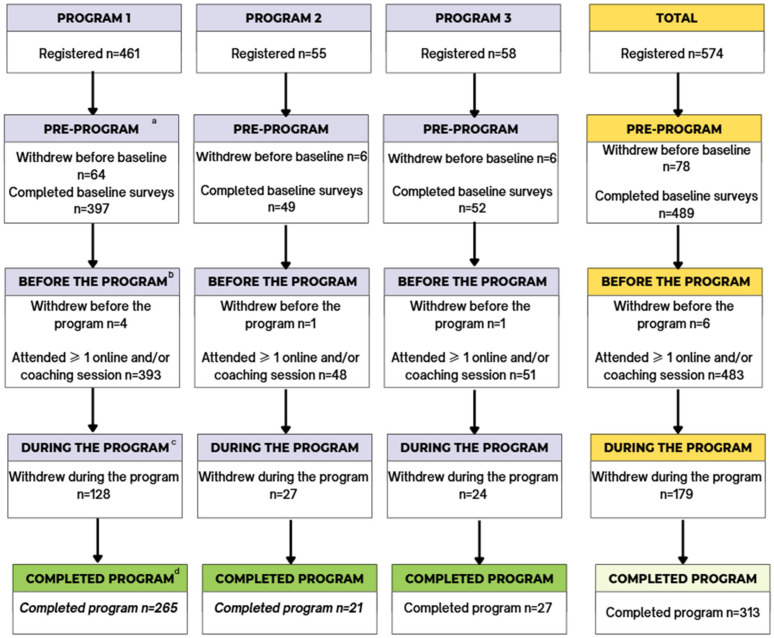
Flow diagram summarizing recruited participants from registration until TEAM program completion. Withdrawal timepoints were categorized as ^a^ registered for program but did not complete any pre-program surveys; ^b^ completed at least one pre-program survey but did not attend or complete any coaching or online sessions; ^c^ completed at least one pre-program survey and at least one online OR coaching session but no post-program surveys; ^d^ completed at least one pre-program and post-program survey and completed or attended at least one online or coaching session.

Between 2018 and 2024 a total of 574 participants registered for the TEAM program, from which 313 participants completed the program and were included in the analysis (Figure 2).

Demographic characteristics are summarized in Table 2, stratified by timepoint of withdrawal. Fifty-six percent of participants included in the final analysis were female (*n* = 176) with a median (IQR) age of 14.4 (13.7–15.8). Most participants resided in areas categorized as higher advantage, with SEIFA deciles of 9 (*n* = 83, 27%) and 10 (*n* = 105, 34%) being the most reported residential postcodes.

Most participants who withdrew from the program did not supply a reason (*n* = 167, 64%). Of those that did, the most commonly reported reason was “not the right time” (*n* = 52, 20%) followed by “no longer interested” (*n* = 20, 8%). There were no significant associations found between demographic characteristics and withdrawal timepoint.

### 3.2. Program Adherence

Attendance data is described in Table 2. Attendance during online modules and coaching sessions was highest amongst participants who completed the program. Most program completers attended all nine online sessions (*n* = 258, 99%) and at least nine of the ten possible coaching sessions (*n* = 231, 74%). The mean ± SD online and coaching sessions attended was 8.6 ± 1.0 and 8.8 ± 1.2, respectively.

Participants who withdrew during the program attended between 0 and 9 online sessions and 0 and 10 coaching sessions, with a mean ± SD attendance of 3.8 ± 3.1 and 4.0 ± 3.2 sessions, respectively. Despite not completing post-program surveys, 25% (*n* = 45) of participants attended seven or more online sessions and 27% (*n* = 48) completed seven or more coaching sessions. Only 14% (*n* = 45) of participants completed at least one survey at the 6-month timepoint; as such, no further analysis of this data was conducted. A descriptive summary of data available from the 6-month follow-up is provided in Supplementary File.

### 3.3. Anthropometry

Pre- and post-program anthropometric data for Program 1 (*n* = 262) and Program 2 (*n* = 19) are summarized in Table 3. There were significant differences in anthropometric outcomes from pre- to post-program (*p* < 0.001) in Program 1 only. Specifically, height and height z-score increased from pre- to post-program, whilst weight and weight z-score reduced over the program. Consequently, reductions in BMI z-scores were observed (median (IQR), with a change in BMI z-score of −0.1 (−0.2, 0.0) (*p* < 0.001). No statistically significant changes were found in anthropometric data from pre- to post-program in Program 2.

### 3.4. Eating Behaviors

**Figure 3:**
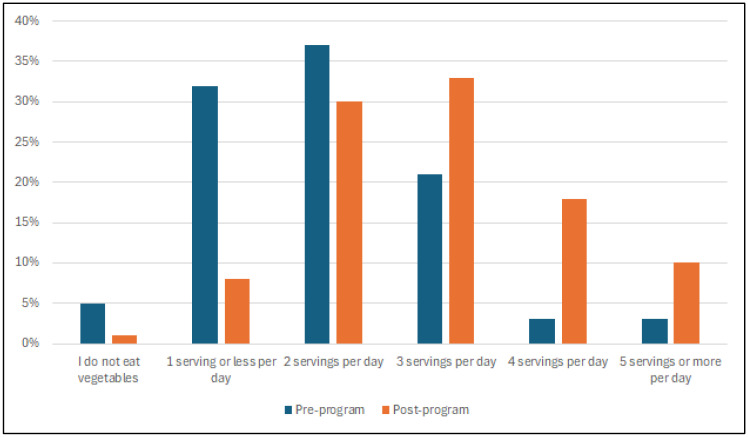
Number of vegetables consumed by participants (%) pre- and post-program based on eating behavior survey responses.

Eating behaviors were self-reported pre- and post-program by 309 (99%) participants (Table 4). Statistically significant associations were found between the pre- and post-program timepoint and consumption of fruit, vegetables and daily water significantly. Fruit consumption recommendations (two servings daily) as per the Australian Guide to Healthy Eating [28] were met by most participants, with 88% eating ≥ two servings of fruit daily post-program compared to 60% pre-program. The number of participants who met consumption of the recommended servings of vegetables (5 for females, 5 ½ for males) [28] increased from 3% to 10% (Figure 3). Water consumption saw a substantial increase, as participants consuming ≥4 cups of water per day increased from 46% pre-program to 70% post-program. Statistically significant associations were also found between pre- and post-program and unhealthy eating behaviors. Portions consumed of take-away food, snacks, confectionery and crisps were reduced across the program, as well as the number of days spent watching screens while consuming meals. Participants who consumed take-away food “never or rarely”/”less than once a week” increased from 56% to 77%, while participants who consumed sweet/savory snacks (e.g., cakes and biscuits) at least once a day reduced from 24% to 6%.

### 3.5. Physical Activity

**Figure 4:**
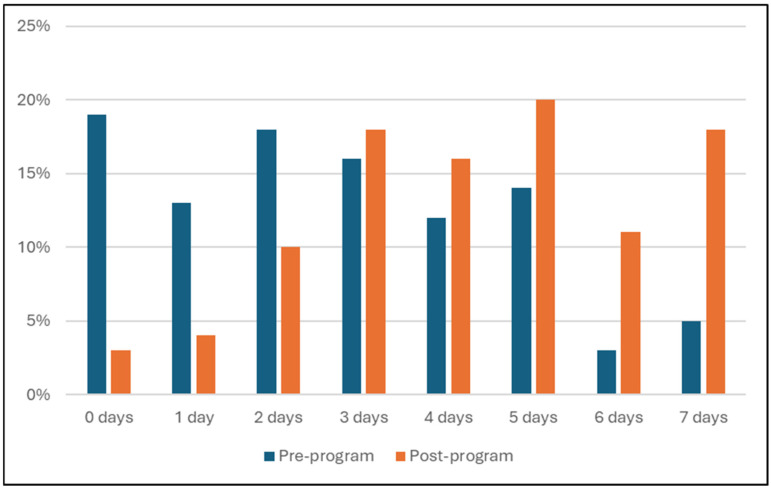
Number of days being physically active per week by participants (%) pre-program and post-program based on physical activity survey responses.

Physical activity surveys were completed by 253 (81%) participants both pre- and post-program (Table 5). Statistically significant associations were found between pre- and post-program and physical activity; days spent exercising per week increased across the program (Figure 4). A shift towards more physically active days was observed, as the number of participants who reported exercising 0 days of the week reduced from 19% to 3% from pre- to post-program. Simultaneously, statistically significant associations were found between pre- and post-program and daily screen time hours; fewer hours were spent watching screens across all days of the week post-program. The greatest decrease in screen time was observed on Saturdays, where there was a decrease from 62% pre- to 33% post-program of participants who reported using screens for more than 3 h daily.

### 3.6. Knowledge and Confidence

Knowledge question surveys were completed by 190 (61%) participants both pre- and post-program (Table 6). Statistically significant results indicated an association between pre- and post-program and number of correct responses. The mean total correct responses increased significantly from 2.58 (±1.2) to 3.58 (±1.0) from pre- to post-program (paired *t*-test, *p* < 0.001).

**Figure 5:**
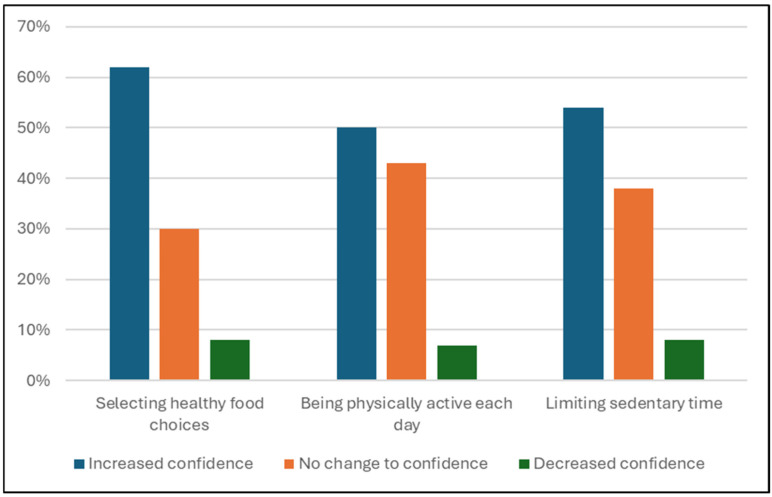
Confidence of healthy food choices, physical activity and sedentary time by participants (%) from pre-program to post-program based on confidence survey responses.

Confidence levels were also assessed in conjunction with the knowledge quiz. A statistically significant association was found between pre- and post-program timepoint and confidence, with post-program confidence scores higher than pre-program (Table 6). Despite overall increased confidence among participants, there were some participants who reported both decreased and unchanged confidence across the three assessed areas (Figure 5).

### 3.7. Wellbeing

As the program evolved there was a change in the surveys administered to assess wellbeing and self-esteem (Table 1). Of the participants, 231 (74%) completed pre- and post-program surveys for both the Body Esteem Scale for Adolescents and Adults and Rosenberg Self and Body Esteem. The Body Esteem Scale for Adolescents and Adults mean ± SD score increased from pre- to post-program from 36.8 ± 17.9 to 47.0 ± 18.3 (*p* < 0.001). Likewise, an increase was observed in the Rosenberg Self- and Body Esteem survey, with the mean ± SD increasing from 17.1 ± 6.8 to 19.4 ± 6.6 (*p* < 0.001).

All participants across the three programs who completed the Wellbeing survey were grouped together for the purpose of analysis (*n* = 80, 26%). A significant association between pre- and post-program and questions relating to feeling “cheerful” (*p* = 0.005) and feeling “fresh and rested” (*p* = 0.010) was found. Participants reported higher ratings of positivity at the post-program timepoint. While there was an increase in the proportion of participants who rated more positively feeling “calm and relaxed”, “active and vigorous” and “daily life has been filled with things that interest me”, no significant associations were found.

## 4. Discussion

This retrospective cohort study evaluated the outcomes (anthropometric measurements, healthy eating behaviors, physical activity, nutrition knowledge, confidence and wellbeing scores) across three cohorts who took part in the TEAM Program from 2018 to 2024. Overall, there were significant associations across all outcomes from pre- to post-program. Significant reductions in BMI, BMI z-score, weight and weight z-scores were reported in Program 1 only. There was an increase in healthy eating behaviors and a decrease in unhealthy eating behaviors, as well as an increase in days per week that participants engaged in moderate–vigorous physical activity and a decrease in sedentary hours per week. Participants’ knowledge, confidence in health behaviors and wellbeing indicators increased from pre- to post-program.

The findings from the evaluation of the TEAM program align closely with established evidence on the effective elements of interventions for child and adolescent obesity, as outlined by the American Academy of Pediatrics [29]. The high-intensity structure of TEAM, featuring eight weekly coaching sessions and interactive online modules, reflects research showing that greater contact hours are associated with stronger treatment effects [29]. TEAM’s multicomponent approach, which integrates nutrition education, physical activity promotion and behavioral strategies, corresponds with evidence supporting comprehensive interventions to reduce BMI in young people [29]. The use of motivational interviewing within coaching sessions supports behavior change and has been linked to improvements in weight status in adolescents [29]. Finally, the program’s family-based design, with flexible delivery tailored to family life and active parental involvement, reinforces the role of the family unit in sustaining healthy behaviors [29,30,31]. Interestingly, the Academy highlights that most interventions included in the evidence summary were conducted face-to-face, calling for more research into alternative modes of intervention delivery. The successful use of e-Health modalities in TEAM contributes important evidence regarding the use of digital platforms for effective management of adolescents with overweight and obesity.

The changes in anthropometric measures in Program 1 point to the effectiveness of TEAM in improving participants’ BMI z-scores from obesity (z-scores ≥ 1.645) to overweight (z-scores 1.036 to 1.645). The significance of the changes was easier to identify with the larger sample size. In comparison, Program 2, with a sample size of 19, saw no significant changes in their anthropometric measures. However, this is to be expected, as the participants’ pre-program BMI z-scores were already within the healthy range, as BMI was no longer included as an eligibility criterion for Program 2. Significant decreases in BMI z-score may be unfavorable in this group. The changes in anthropometric measures in Program 1 align with existing evidence suggesting that e-Health interventions are effective at reducing BMI and BMI z-scores [32,33,34]. The TEAM program involved weekly tele-health sessions. Weekly tele-health sessions were also found to be effective in achieving weight loss in study participants who took part in a randomized controlled trial (RCT) that used weekly sessions as an intervention for weight loss [35]. The participants who took part in the TEAM program were aged 14–17 years old; the results indicated that it is also possible that BMI scores may have improved due to the participants maintaining their weight while continuing to grow in length, which is in line with current recommendations [29,30].

Eating behaviors improved across all cohorts. Current evidence-based practice guidelines support family-based interventions [29,31,36]; the behaviors may have been easier to reinforce due to the family-centered administration of the TEAM program and the age of the participants, who as adolescents may have limits to the level of control over food choice/watching television while eating meals at home. Across all programs, participants increased their consumption of fruits, vegetables and water. These findings align with a 2025 systematic review exploring the use of digital interventions to promote healthy eating behavior in adolescents [19]. Whilst the review yielded mixed results regarding intervention adherence and effectiveness, digital interventions that employed behavior change techniques similar to TEAM, such as goal setting and social support, may be associated with increased effectiveness [19].

Although nutrition knowledge scores increased across the cohort, the results show that knowledge gaps remained regarding recommended vegetable intake and screen time. For example, recommendations for vegetable intake and screen time were correctly identified by only 38% and 57% of the respondents post-program, respectively. This is compared to >80% in other knowledge questions. However, self-reported screen time significantly reduced, and vegetable consumption significantly increased. This indicates that even though the knowledge was lacking (despite knowledge scores increasing), the healthy behaviors improved. Gamification could be utilized in the design of future versions of the program. E-Health interventions aimed at improving nutrition behaviors that incorporate gamification to encourage behavior change were found to enhance participants’ nutrition knowledge and self-efficacy in the short term [37].

Physical activity levels significantly increased and self-reported screen time significantly decreased from pre-program to completion of the e-Health program. A recent systematic review and meta-analysis reported that physical activity levels improved for adolescents when interventions used mobile phone text messaging to deliver motivational and informative messages [38]. Improvements were also seen in screen time reductions, which may be a feature that could be incorporated into future designs of the TEAM program.

Participants’ confidence increased, with 50–62% of participants reporting increased confidence in all three assessed areas: making healthy food choices, physical activity levels and sedentary time. While not all participants reported increased confidence, the applied health behaviors show the increasing capability amongst participants. Increased self-esteem and confidence help to facilitate positive behavior change, which is required for long-term weight management [39]. Participants’ wellbeing scores improved from pre-program to program completion. Similar findings were observed in a 2024 systematic review, which found that there is strong evidence that e-Health interventions positively impact mental health/wellbeing when the following elements are present: self-monitoring and peer or parental involvement [40].

This evaluation adds to the current evidence base on the effectiveness of e-Health interventions for eliciting positive behavior changes in the short term; however, more research is needed to investigate long-term behavior change [41]. More research also needs to be completed to determine which mode of e-Health delivery is most beneficial (hybrid, online or mixed online and mobile phone messaging) [41]. E-Health interventions that included tailored feedback to participants and also incorporate gamification have been found to be more effective in eliciting positive dietary behavior changes, particularly increased fruit and vegetable consumption [19]. The TEAM Program used incentives and tokens to encourage positive behavior change among participants.

Participants in the study predominantly resided in areas of higher socio-economic advantage; this limits the generalizability of the findings to adolescents residing in areas of lower socio-economic advantage. There is a significant gap in the literature regarding effective digital health interventions for obesity and health outcomes that address equity [42]. Further study is required to support the development of more equitable e-Health programs targeted at adolescents so that the disparities in obesity and health outcomes can be addressed. More research needs to focus on experiences of families from more diverse backgrounds who take part in e-Health programs [43]. Withdrawal rates and reasons were similar across the different areas of socio-economic advantage. Co-designing interventions with end users (adolescents), personalization and just-in-time adaptations improves effective engagement with digital interventions and could reduce withdrawal rates in future programs [44]. Smart phone capabilities, such as messaging and apps, are associated with high acceptability of interventions and reduced withdrawal rates [45,46]; the TEAM program did not engage smartphone capabilities, so this could be further explored to reduce withdrawal rates in future programs. Evidence around mobile health applications for children and adolescents with obesity has shifted focus recently from message to app usage, reflecting changes in how young people engage with smartphone technology [47].

Other limitations of the study include the use of bespoke surveys developed by the administering organization. Future evaluation should include the use of validated nutrition and physical activity assessments. Anthropometric measurements were self-reported, which could lead to possible errors or social desirability bias; however, an Australian study demonstrated that parents are relatively accurate reporters of these measures [48]. This study is also a retrospective evaluation, so causal relationships cannot be produced.

Despite these limitations, completion of the TEAM e-Health program was associated with positive changes in anthropometry and health behaviors. The TEAM program is one of the few multicomponent interventions available for adolescents, with many interventions targeting only a single health outcome [41]. Focusing on behavior change, increasing confidence, knowledge and wellbeing, rather than a purely weight-centric focus, was a strength of the program. These positive changes support the notion of improved cost effectiveness when delivering public health programs via e-Health compared to conventional face-to-face programs. A recent cost-effectiveness analysis of a hypothetical e-Health intervention for adolescents with overweight and obesity determined that the incremental cost effectiveness of the e-Health intervention was dominant—indicating it was cheaper and more effective [49]. Regarding the TEAM program, the education was delivered to families via a web platform, significantly reducing the resources required to deliver education via in-person sessions. The contact time with the health coaches (still less than what would be required for in-person sessions) could then be focused on individualized goal setting to facilitate the implementation of behavior change. The improved cost effectiveness and accessibility—particularly in terms of reach and availability—highlight the potential for e-Health interventions to be scaled up and delivered to young people and families around Australia.

## 5. Conclusions

This study provides a unique Australian perspective on the effectiveness of e-Health interventions for adolescents. Participation in the TEAM program was associated with reductions in anthropometric measures and improvements in health behaviors, including increased physical activity, healthier food choices and enhanced wellbeing. These findings support the potential of digital health programs to promote positive lifestyle changes among adolescents, particularly outside school-based settings. A key limitation of this evaluation was the lack of long-term follow-up data. Future research should explore the sustainability of behavior changes achieved by the TEAM program and evaluate the relative effectiveness compared to other e-Health approaches.

## Figures and Tables

**Table 1:** Outcome measures collected via online surveys.

Outcome Measure	Description
Anthropometry (Program 1 and 2 only)	Standing height (cm) and weight (kg) were measured either by a caregiver or a health professional using their own scales. BMI was calculated as weight/height(m)^2^. Measurements were converted into a z-score using reference data from Centers for Disease Control and Prevention [25].
Eating Habits	Ten-question multiple choice survey assessing weekly dietary intake including drinks, snacks, discretionary food items and meals eaten at night in front of the television (qualitative assessment only).
Physical Activity	Six-question survey consisting of four multiple choice and two open-answer questions about physical activity and sedentary habits during the week.
Knowledge Questions	Five-question multiple choice survey based on current knowledge and feelings towards nutrition and PA and confidence relating to healthy eating habits and being physically active.
Self-Esteem and Body Esteem (Program 1 only)	“Body Esteem Scale for Adolescents and Adults” Medelson and White. This tool assesses general feelings about an individual’s current appearance, weight satisfaction and body appearance [26]. The survey consists of 23 questions on a 5-point Likert scale. Each question receives a score between 0 and 4 for a maximum total score of 92. Higher scores indicate a higher level of self- and body esteem.
Self-Perception (Program 1 only)	Adaptation of the Rosenberg Self-Esteem Scale [27], a validated tool that allows respondents to reflect on self-perception. The tool was altered to suit the age demographic of participants. The survey consists of ten questions on a 4-point Likert scale, each question scoring between 0 and 3 for a maximum score of 30. A higher score indicates a higher level of self- and body esteem.
Wellbeing	The Wellbeing survey consists of five questions on a 6-point Likert scale asking respondents to reflect on their quality of life and emotions over the past two weeks.

**Table 2:** Pre-program demographic characteristics of participants and compliance of TEAM program stratified by withdrawal timepoint.

	Withdrew Before Pre-Program ^a^	Withdrew Before the Program ^b^	Withdrew During the Program ^c^	Completed Program ^d^	*p*-Value
n = 574	76	6	179	313	n/a
Age at start, median (IQR)	15.0 (13.8–15.7)	14.5 (13.3–15.4)	14.5 (13.6–15.9)	14.4 (13.7–15.8)	0.77 ^e^
Sex at birth					
Female	32 (42)	3 (50)	101 (56)	176 (56)	0.15 ^f^
Male	44 (58)	3 (50)	78 (44)	137 (44)	
Referring Health Professional					
Dentist	0 (0)	0 (0)	0 (0)	1 (0)	n/a
Dietitian/nutritionist	2 (3)	0 (0)	5 (3)	8 (3)	
GP/doctor	5 (7)	0 (0)	10 (6)	28 (9)	
Medical/surgical specialist	4 (5)	0 (0)	12 (7)	18 (6)	
Nurse	3 (4)	0 (0)	19 (11)	21 (7)	
Other ^g^	2 (3)	0 (0)	1 (1)	4 (1)	
Not specified	60 (79)	6 (100)	132 (73)	233 (74)	
SEIFA decile *n* = 567					
1	5 (7)	0 (0)	10 (6)	11 (42)	0.21 ^f^
2	2 (3)	0 (0)	11 (6)	10 (3)	
3	3 (4)	0 (0)	14 (8)	10 (3)	
4	7 (9)	1 (17)	15 (8)	15 (5)	
5	4 (5)	0 (0)	6 (3)	7 (2)	
6	0 (0)	0 (0)	1 (1)	0 (0)	
7	8 (11)	1 (17)	21 (12)	32 (10)	
8	12 (16)	2 (33)	20 (11)	35 (11)	
9	13 (17)	0 (0)	28 (16)	83 (27)	
10	21 (28)	2 (33)	52 (29)	105 (34)	
Withdrawal reason					
Time/location does not suit the participant	1 (1)	0 (0)	3 (2)	-	n/a
No longer interested	7 (9)	0 (0)	13 (7)	-	
Medical reasons	4 (5)	0 (0)	2 (1)	-	
Not the right time	19 (25)	1 (17)	32 (18)	-	
Ineligible due to BMI criteria ^h^	1 (1)	0 (0)	7 (4)	-	
Planning to engage in future program	1 (1)	0 (0)	3 (2)	-	
Reason not specified	43 (57)	5 (83)	118 (66)	-	
Attendance (mean ± SD)					n/a
Online sessions ^j^	-	-	3.8 ± 3.1	8.6 ± 1.0	
Coaching sessions ^k^	-	-	4.0 ± 3.2	8.8 ± 1.2	

Data reported as n (%) unless specified. *p*-value describes significance of associations between demographic variable and withdrawal timepoint. Statistical significance *p* < 0.05. ^a^ Registered for program but did not complete any pre-program surveys; ^b^ completed at least one pre-program survey but did not attend or complete any coaching or online sessions; ^c^ completed at least one pre-program survey and at least one online OR coaching session but no post-program surveys; ^d^ completed at least one pre-program and post-program survey and completed or attended at least one online or coaching session; ^e^ Kruskal–Wallis test; ^f^ Chi-square test; ^g^ other health professional includes occupational therapist, physiotherapist, psychologist/counsellor or social worker; ^h^ only applicable to Program 1 participants; ^j^ total number of online sessions in the program = 9; ^k^ total number of coaching sessions in the program = 10.

**Table 3:** Pre-program and post-program anthropometric data for participants in TEAM Program 1 and Program 2.

	Program 1 (*n* = 262)				Program 2 (*n* = 19) ^a^			
	Pre-Program	Post-Program	Change	*p*-Value	Pre-Program	Post-Program	Change	*p*-Value
BMI (kg/m^2^)	28.6 (25.3, 32.5)	27.7 (24.2, 31.6)	−0.8 (−1.7, −0.1)	<0.001	21.6 (18.5, 28.6)	22.2 (18.9, 27.7)	0.0 (−0.6, 0.9)	0.97
Height (cm)	164.3 (158.5, 170.5)	165.0 (160.0, 172.0)	1.0 (0.0, 2.0)	<0.001	165.0 (156.0, 172.0)	165.0 (158.0, 172.0)	0.0 (−1.0, 2.0)	0.59
Weight (kg)	78.0 (65.5, 92.0)	77.0 (64.0, 90.0)	−1.0 (−3.1, 0.5)	<0.001	60.0 (50.0, 71.0)	56.0 (52.0, 73.0)	0.0 (−1.0, 2.0)	0.47
BMI z-score	1.75 (1.4, 2.2)	1.65 (1.1, 2.1)	−0.10 (−0.2, 0.0)	<0.001	0.87 (−0.3, 1.6)	1.03 (0.1, 1.6)	0.16 (−0.2, 0.1)	0.71
Height z-score	0.22 (−0.5, 1.1)	0.36 (−0.4, 1.3)	0.14 (−0.0, 0.2)	<0.001	0.71 (−0.2, 1.3)	0.30 (−0.2, 1.4)	−0.41 (−0.2, 0.1)	0.98
Weight z-score	1.79 (1.2, 2.3)	1.70 (1.0, 2.2)	−0.09 (−0.2, 0.0)	<0.001	0.63 (0.2, 1.9)	0.65 (0.2, 2.1)	0.02 (−0.2, 0.1)	0.84

Data reported as median (IQR). *p*-Value comparing pre-program and post-program result using Wilcoxon signed rank test. Statistical significance *p* < 0.05. ^a^ Program 2 eligibility criteria did not include BMI. Program 3 participants did not complete anthropometric data.

**Table 4:** Change in eating behaviors from pre- to post-program in participants completing the TEAM program (*n* = 309).

		Pre-Program	Post-Program	*p*-Value
Fruit	I do not eat fruit	16 (5)	2 (1)	<0.001
	1 serving or less per day	109 (35)	36 (12)	
	2 servings per day	121 (39)	171 (55)	
	3 servings per day	51 (17)	73 (24)	
	4 servings or more per day	12 (4)	27 (9)	
Vegetables	I do not eat vegetables	14 (5)	2 (1)	<0.001
	1 serving or less per day	99 (32)	25 (8)	
	2 servings per day	113 (37)	92 (30)	
	3 servings per day	64 (21)	103 (33)	
	4 servings per day	10 (3)	57 (18)	
	5 servings or more per day	9 (3)	30 (10)	
Water	I do not drink water	1 (0)	0 (0)	<0.001
	Less than 1 cup per day	9 (3)	3 (1)	
	1–2 cups per day	33 (11)	7 (2)	
	2–3 cups per day	59 (19)	29 (9)	
	3–4 cups per day	66 (21)	53 (17)	
	4 cups or more	141 (46)	217 (70)	
Soft drink	I do not drink soft drink	70 (23)	107 (35)	n/a ^a^
	Less than 1 cup per week	100 (32)	112 (36)	
	1–3 cups per week	95 (31)	68 (22)	
	4–6 cups per week	22 (7)	22 (7)	
	1–2 cups per day	14 (5)	6 (2.0)	
	2–3 cups per day	2 (1)	1 (0)	
	3 or more cups per day	6 (2)	3 (1)	
Fried potato	Never or rarely	38 (12)	80 (26)	n/a ^a^
	Less than once a week	117 (38)	129 (42)	
	1–2 times a week	115 (37)	89 (29)	
	3–4 times a week	30 (10)	10 (3)	
	5–6 times a week	4 (2)	0 (0)	
	Once a day	3 (1)	1 (0)	
	2 or more times a day	1 (0)	0 (0)	
Take-away	Never or rarely	64 (21)	94 (30)	<0.001
	Less than once a week	104 (34)	143 (46)	
	1–2 times a week	114 (37)	65 (21)	
	3–4 times a week	23 (7)	7 (2)	
	5–6 times a week	2 (1)	0 (0)	
	Once a day	2 (1)	0 (0)	
	2 or more times a day	0 (0)	0 (0)	
Meal while watching TV	Never or rarely	179 (58)	204 (66)	0.005
	1 day a week	25 (8)	32 (10)	
	2 days a week	14 (5)	20 (7)	
	3 days a week	24 (8)	16 (5)	
	4 days a week	7 (2)	9 (3)	
	5 days a week	10 (3)	10 (3)	
	6 days a week	7 (2)	2 (1)	
	7 days a week	43 (14)	16 (5)	
Sweet/savory snacks	Never or rarely	24 (8)	47 (15)	<0.001
	Less than once a week	45 (15)	101 (33)	
	1–2 times a week	79 (26)	94 (30)	
	3–4 times a week	59 (19)	37 (12)	
	5–6 times a week	29 (9)	11 (4)	
	Once a day	34 (11)	14 (5)	
	2 or more times a day	39 (13)	5 (2)	
Confectionery	Never or rarely	36 (11)	71 (23)	<0.001
	Less than once a week	71 (23)	115 (37)	
	1–2 times a week	102 (33)	92 (30)	
	3–4 times a week	22 (7)	21 (7)	
	5–6 times a week	55 (18)	5 (2)	
	Once a day	22 (7)	4 (1)	
	2 or more times a day	17 (6)	1 (0)	
Crisps	Never or rarely	34 (11)	74 (24)	<0.001
	Less than once a week	76 (25)	113 (37)	
	1–2 times a week	100 (32)	83 (27)	
	3–4 times a week	62 (20)	26 (8)	
	5–6 times a week	13 (4)	9 (3)	
	Once a day	14 (5)	3 (1)	
	2 or more times a day	10 (3)	1 (0)	

Data reported as n (%). Statistical significance: *p* < 0.05. *p*-Value determined using Chi-square test. **^a^** Assumptions of Chi-squared not met, unable to run statistical analysis.

**Table 5:** Change in physical activity behaviors from pre-program to post-program in participants completing the TEAM program (*n* = 253).

			Pre-Program	Post-Program	*p*-Value
Over the past 7 days, on how many days did you participate in moderate to vigorous exercise?		0 days	58 (19	10 (3)	<0.001
		1 day	38 (13)	12 (4)	
		2 days	53 (18)	30 (10)	
		3 days	49 (16)	55 (18)	
		4 days	36 (12)	48 (16)	
		5 days	43 (14)	60 (20)	
		6 days	8 (3)	32 (11)	
		7 days	15 (5)	53 (18)	
Time spent using a mobile phone, iPad, tablet, computer, gaming console or watching TV/DVD ^a^	School day	0–1 h	18 (6)	55 (18)	<0.001
		1–2 h	80 (27)	133 (44)	
		2–3 h	73 (24)	63 (21)	
		>3 h	129 (43)	49 (16)	
	Saturday	0–1 h	8 (3)	23 (8)	<0.001
		1–2 h	38 (13)	87 (29)	
		2–3 h	68 (23)	90 (30)	
		>3 h	186 (62)	100 (33)	
	Sunday	0–1 h	9 (3)	30 (10)	<0.001
		1–2 h	38 (13)	93 (31)	
		2–3 h	85 (28)	82 (27)	
		>3 h	168 (56)	95 (32)	

Data reported as n (%). Data statistical significance: *p* < 0.05. *p*-value determined using Chi-square test. ^a^ Excludes time at school or doing homework.

**Table 6:** Change in knowledge and confidence from pre- to post-program in participants completing the TEAM program (*n* = 190).

		Pre-Program	Post-Program	*p*-Value
**Knowledge Questions ^a^**				
How many meals and snacks should I aim to have each day for healthy, regular eating?		143 (75)	172 (91)	0.001
How many servings of vegetables are recommended that I eat each day?		46 (24)	72 (38)	0.004
When reading nutrition information panels, what sort of information should I look out for?		102 (54)	165 (87)	<0.001
How many minutes of physical activity is it recommended that I do each day?		123 (65)	162 (85)	<0.001
How much screen time is it recommended that I stick to each day?		76 (40)	108 (57)	0.001
Total correct (mean ± SD) ^b^		2.58 ± 1.2	3.58 ± 1.0	<0.001 ^c^
**Confidence**				
I feel confident in selecting healthy food choices	Strongly agree	24 (13)	67 (35)	<0.001
	Agree	70 (37)	99 (52)	
	Neither agree nor disagree	71 (37)	20 (11)	
	Disagree	18 (10)	3 (2)	
	Strongly disagree	7 (4)	1 (1)	
I feel confident being physically active each day	Strongly agree	30 (16)	65 (34)	<0.001
	Agree	69 (36)	78 (41)	
	Neither agree nor disagree	47 (25)	34 (18)	
	Disagree	34 (18)	11 (6)	
	Strongly disagree	10 (5)	2 (1)	
I feel confident limiting the amount of time I spend being sedentary	Strongly agree	13 (7)	13 (7)	0.001
	Agree	57 (30)	75 (40)	
	Neither agree nor disagree	65 (34)	53 (28)	
	Disagree	35 (18)	13 (7)	
	Strongly disagree	20 (11)	7 (4)	

Data reported as n (%) unless specified. Data statistical significance: *p* < 0.05. *p*-Value determined using Chi-square test unless specified. **^a^** Indicated number of participants who responded correctly to the question. ^b^ Total correct score out of 5 questions. ^c^ *p*-Value determined by a paired *t*-test.

## Data Availability

The datasets presented in this article are not readily available because ethics approval was obtained only for the evaluation of anonymized data for the purpose of this research only. Requests to access the datasets should be directed to the Better Health Company.

## Supplementary Materials

The following supporting information can be downloaded at https://www.mdpi.com/article/10.3390/nu17223509/s1, Table S1. Pre-program demographic characteristics of TEAM participants who completed at least at least 1 follow up assessment at 6 months post-program. Table S2. Anthropometric data (median, IQR) for participants in TEAM Program 1 and Program 2 at 6 months post-program. Table S3. Eating behaviors in participants completing the TEAM program at 6 months post-program. Table S4. Physical activity behaviors in participants completing the TEAM program at 6 months post-program. Table S5. Knowledge and confidence in participants completing the TEAM program at 6 months post-program. Table S6. Wellbeing outcomes from participants completing the TEAM program at 6 months post-program.

## Abbreviations

The following abbreviations are used in this manuscript:

BMI	Body mass index
IQR	Interquartile range
SD	Standard deviation
SEIFA	Socio-Economic Indexes for Areas
TEAM	Think Eat And Move (program)
