# Supplementary material for: Evaluating the Impact of an 8-Week Family-Focused E-Health Lifestyle Program for Adolescents: A Retrospective, Real-World Evaluation
Source: Nutrients. 2025 Nov 10;17(22):3509. doi: 10.3390/nu17223509 (PMC12655396; doi:10.3390/nu17223509)
Supplement: Supplementary file 1 [file nutrients-17-03509-s001.zip › nutrients-3929728-supplementary.pdf]

Table S1. **Pre-program demographic characteristics of TEAM participants who completed at least at least 1 follow up assessment at 6 months post-program.**

	6-months post-program (n=45)
**Program 1, n (%)**	38 (84)
**Program 2, n (%)**	7 (16)
**Program 3, n (%)**	0 (0)
**Age at start, median (IQR)**	14.9 (14.4-15.7)
**Sex at birth, n (%)**	
Female	34 (76)
Male	11 (24)
**Referring Health Professional occupation, n (%)**	
Dietitian/ nutritionist	1 (2)
GP/Doctor	2 (4)
Medical/ surgical specialist	5 (11)
Nurse	4 (9)
Not specified	33 (73)

Table S2. Anthropometric data (median, IQR) for participants in TEAM Program 1 and Program 2 at 6 months post-program.

	Program 1 6-months post-program (n=32)	Program 2 6-months post-program (n=5)
**BMI (kg/m^2)**	27.6 (22.8, 30.9)	19.5 (18.7, 19.7)
**Height (cm)**	163.7 (159.5, 168.0)	158.0 (157.0, 163.0)
**Weight (kg)**	72.8 (60.0, 83.6)	48.0 (42.0, 55.0)
**BMI z-score**	1.5 (0.8, 2.0)	0.21 (-0.1, 0.3)
**Height z-score**	0.2 (-0.7, 0.2)	0.2 (0.1, 0.2)
**Weight z-score**	1.5 (0.7, 1.9)	0.3 (-0.4, 0.3)

Table S3. Eating behaviors in participants completing the TEAM program at 6 months post-program.

		6-months (n=45) n (%)
**Fruit**	I don't eat fruit	0 (0)
	1 serve or less per day	6 (13)
	2 serves per day	26 (58)
	3 serves per day	10 (22)
	4 serves or more per day	3 (7)
**Vegetables**	I don't eat vegetables	0 (0)
	1 serve or less per day	2 (4)
	2 serves per day	24 (53)
	3 serves per day	11 (24)
	4 serves per day	4 (9)
	5 serves or more per day	4 (9)
**Water**	I don't drink water	1 (2)
	Less than 1 cup per day	0 (0)
	1-2 cups per day	4 (9)
	2-3 cups per day	6 (13)
	3-4 cups per day	8 (18)
	4 cups or more	26 (58)
**Soft drink**	I don't drink soft drink	15 (33)
	Less than 1 cup per week	23 (51)
	1-3 cups per week	5 (11)
	4-6 cups per week	2 (4)
	1-2 cups per day	0 (0)
	2-3 cups per day	0 (0)
	3 or more cups per day	0 (0)
**Fried potato**	Never or rarely	7 (16)
	Less than once a week	25 (56)
	1-2 times a week	10 (22)
	3-4 times a week	3 (7)
	5-6 times a week	0 (0)
	Once a day	0 (0)
	2 or more times a day	0 (0)
**Take-away**	Never or rarely	13 (29)
	Less than once a week	21 (47)
	1-2 times a week	10 (22)
	3-4 times a week	1 (2)
	5-6 times a week	0 (0)
	Once a day	0 (0)
	2 or more times a day	0 (0)

		6-months (n=45)<br>n (%)
**Meal while watching TV**	Never or rarely	27 (60)
	1 day a week	5 (11)
	2 days a week	3 (7)
	3 days a week	4 (9)
	4 days a week	4 (9)
	5 days a week	1 (2)
	6 days a week	0 (0)
	7 days a week	1 (2)
**Sweet/savoury snacks**	Never or rarely	3 (7)
	Less than once a week	16 (36)
	1-2 times a week	17 (38)
	3-4 times a week	8 (18)
	5-6 times a week	0 (0)
	Once a day	1 (2)
	2 or more times a day	0 (0)
**Confectionery**	Never or rarely	6 (13)
	Less than once a week	17 (38)
	1-2 times a week	16 (36)
	3-4 times a week	6 (13)
	5-6 times a week	0 (0)
	Once a day	0 (0)
	2 or more times a day	0 (0)
**Crisps**	Never or rarely	9 (20)
	Less than once a week	18 (40)
	1-2 times a week	15 (33)
	3-4 times a week	3 (7)
	5-6 times a week	0 (0)
	Once a day	0 (0)
	2 or more times a day	0 (0)

Table S4. Physical activity behaviors in participants completing the TEAM program at 6 months post-program.

			6-months (n=42)<br>n (%)
**Over the past 7 days, on how many days did you participate in moderate to vigorous exercise?**		0 days	2 (5)
		1 day	2 (5)
		2 days	6 (14)
		3 days	5 (12)
		4 days	7 (17)
		5 days	9 (21)
		6 days	6 (14)
		7 days	5 (12)
**Time spent using a mobile phone, iPad, tablet, computer, gaming console or watching TV/DVD** [a]	School day	0-1 hour	8 (19)
		1-2 hours	15 (36)
		2-3 hours	9 (21)
		> 3 hours	10 (24)
	Saturday	0-1 hour	1 (2)
		1-2 hours	17 (38)
		2-3 hours	14 (33)
		> 3 hours	10 (24)
	Sunday	0-1 hour	6 (14)
		1-2 hours	12 (29)
		2-3 hours	17 (40)
		> 3 hours	7 (17)

Table S5. Knowledge and confidence in participants completing the TEAM program at 6 months post-program.

		(n=25) n (%)
Knowledge Questions		
How many meals and snacks should I aim to have each day for healthy, regular eating?		22 (88)
How many serves of vegetables are recommended that I eat each day?		10 (40)
When reading nutrition information panels, what sort of information should I look out for?		14 (56)
How many minutes of physical activity is it recommended that I do each day?		19 (76)
How much screen time is it recommended that I stick to each day?		10 (40)
Confidence		**(n=25) n (%)**
**I feel confident in selecting healthy food choices**	Strongly agree	6 (24)
	Agree	15 (60)
	Neither agree nor disagree	4 (16)
	Disagree	0 (0)
	Strongly disagree	0 (0)
**I feel confident being physically active each day**	Strongly agree	8 (32)
	Agree	12 (48)
	Neither agree nor disagree	3 (12)
	Disagree	2 (8)
	Strongly disagree	0 (0)
**I feel confident limiting the amount of time I spend being sedentary**	Strongly agree	3 (12)
	Agree	10 (40)
	Neither agree nor disagree	10 (40)
	Disagree	2 (8)
	Strongly disagree	0 (0)

Table S6. Wellbeing outcomes from participants completing the TEAM program at 6 months post-program.

**Self and body esteem scales (n=33), mean ± SD**	
Body Esteem Scale for Adolescents and Adults	45.4 ± 14.9
Rosenberg Self and Body Esteem survey	20.7 ± 4.4
**Wellbeing Questionnaire (n=8), n (%)[a]**	
Feeling…	
Cheerful	7 (88)
Active	5 (63)
Fresh and rested	7 (88)
Filled with interest	1 (13)
Calm and relaxed	7 (88)

[a] *For the Wellbeing Survey, participants responded on a 6-point scale. Scores 1-3 correspond to a negative response, whilst scores 4-6 correspond to a positive response. Positive responses are indicated in this table.*
